# Myeloid sarcoma of the septum pellucidum causing hallucinations and personality changes in an elderly woman: a case report

**DOI:** 10.1007/s00277-026-07127-5

**Published:** 2026-06-11

**Authors:** Rodrigo Garcia-Santisteban, Maria Kaufman, Monica Vinay, Hac-Tu Jacqueline Chung, Warren Brenner, Vlad Voin

**Affiliations:** 1 School of Medicine, Department of Internal Medicine, Florida Atlantic University, Boca Raton, FL USA; 2 Miller School of Medicine, University of Miami, Miami, FL USA; 3 School of Medicine, Anahuac University, Mexico City, Mexico; 4 College of Medicine, Department of Infectious Disease, University of Florida, Gainesville, FL USA; 5 Department of Hematology/Oncology, Lynn Cancer Center, Boca Raton, FL USA

**Keywords:** Myeloid sarcoma, Acute myeloid leukemia, Visual hallucinations, Central nervous system neoplasms, Case report

## Abstract

Myeloid sarcoma is an extramedullary tumor composed of myeloid blasts, most commonly involving the skin, lymph nodes, and soft tissues. We report a rare case of myeloid sarcoma of the central nervous system (CNS) causing neuropsychiatric symptoms in an elderly woman with a history of JAK2-positive polycythemia vera and acute myeloid leukemia (AML) in long-term remission. The patient presented with a one-month history of complex visual hallucinations, short-term memory loss, and personality changes. Magnetic resonance imaging revealed a 2.4-cm enhancing mass in the septum pellucidum extending between the frontal horns of the lateral ventricle. Cytological analysis of the cerebrospinal fluid showed pleocytosis, and flow cytometry identified a 50% population of CD34 + blasts, consistent with the immunophenotype of the patient’s prior biopsy-proven AML. This case highlights an exceedingly unusual presentation of AML relapse and emphasizes the need for clinicians to consider CNS involvement in hematologic patients presenting with new-onset neuropsychiatric symptoms, regardless of remission status.

## Introduction

Elderly patients commonly present with vague clusters of neuropsychiatric symptoms that may be caused by multiple systemic pathological processes, often posing diagnostic challenges for clinicians [1]. Myeloid sarcoma (MS) is a rare manifestation of acute myeloid leukemia (AML), typically found in the skin, lymph nodes, and soft tissues; however, intra-axial central nervous system (CNS) localization is exceedingly rare and carries a complex prognosis [2–6]. While intracranial manifestations are documented, they often mimic other CNS pathologies, and the lack of standardized guidelines makes treatment outcomes and prognoses highly variable [4, 6]. We present the unique case of an 88-year-old woman whose AML relapse manifested as an isolated tumor of the septum pellucidum, presenting with profound personality changes, visual hallucinations, memory loss, and depressive symptoms.

## Case presentation

An 88-year-old woman with a past medical history of JAK2-positive polycythemia vera (PV) with transformation to AML in long-term remission on modified azacitidine/venetoclax presented to the emergency department with a 1-month history of complex visual hallucinations, short-term memory loss, and depressive symptoms.

The patient had been followed in the Hematology/Oncology clinic for 5 years since her diagnosis of PV and subsequent AML. At the time of her initial AML diagnosis, bone marrow biopsy with flow cytometry demonstrated a population of CD34 + blasts, and next-generation sequencing (NGS) had identified somatic mutations in TET2, EZH2, ASXL1, BCOR, and STAG2. Cytogenetics was not performed at that time. She had been living independently and had multiple prior negative screenings for depression. However, her daughter reported that over the past month, the patient developed decreased interest in hobbies and family interactions, significant difficulty remembering recent events, and intermittent disorientation to place and time. The patient also reported episodes of anxiety-provoking, paranoid visual hallucinations. She denied fevers, chills, cough, vomiting, diarrhea, or illicit drug use.

**Fig. 1:**
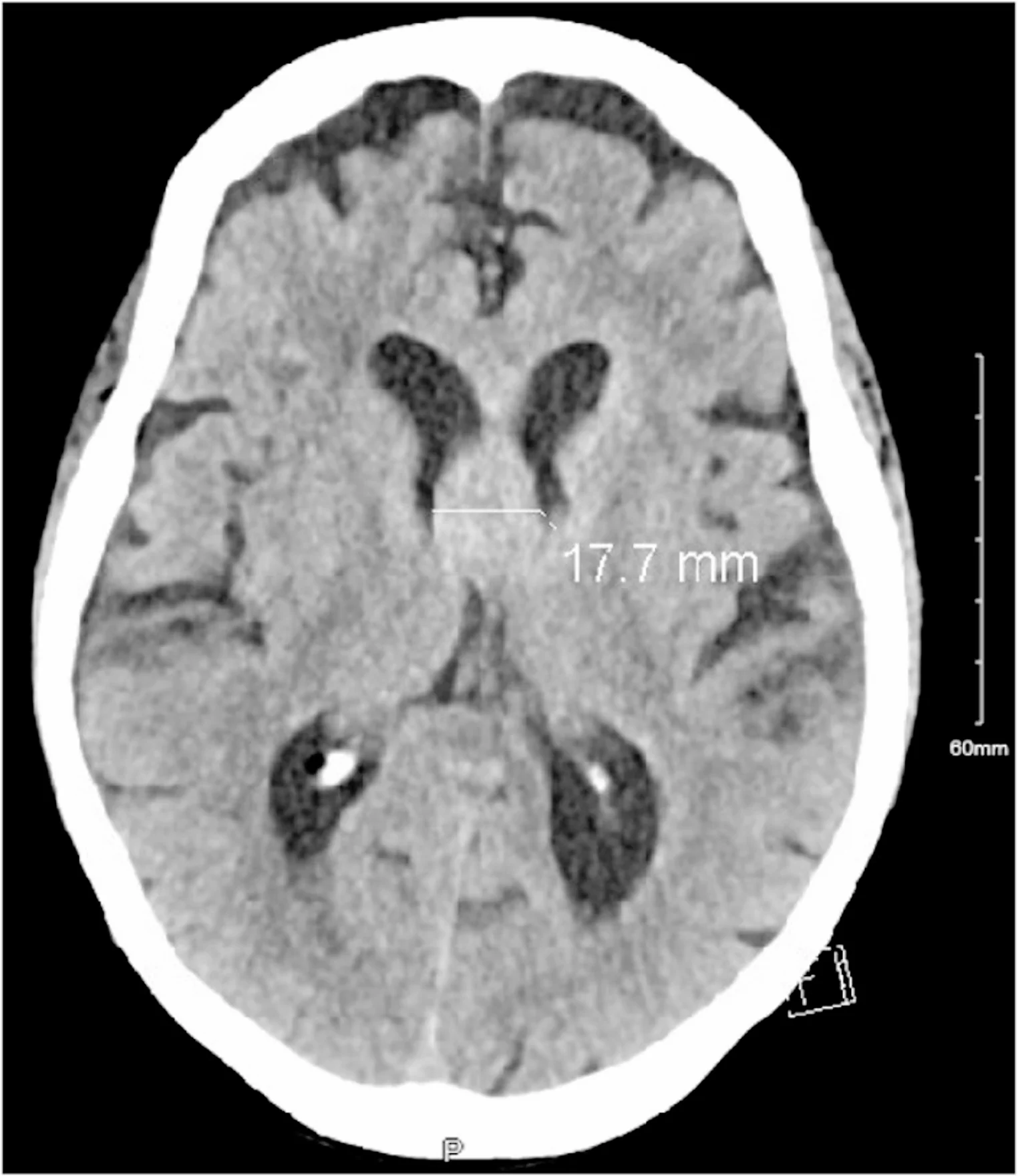
Axial view of a simple computed tomography of the brain showing diffuse thickening of the interventricular septum without hydrocephalus

On presentation, vital signs were within normal limits. Neurological examination showed orientation to person and place, but not to time. Deep tendon reflexes were preserved and symmetrical, and cranial nerve function was intact. Laboratory testing was notable for mild leukocytosis (12.8 K/µL) without peripheral blasts. Urinalysis was negative for infection. Electroencephalography (EEG) showed generalized theta-range slowing, consistent with an encephalopathic process. A non-contrast computed tomography (CT) scan of the brain demonstrated diffuse thickening of the interventricular septum without hydrocephalus (Fig. 1).

**Fig. 2:**
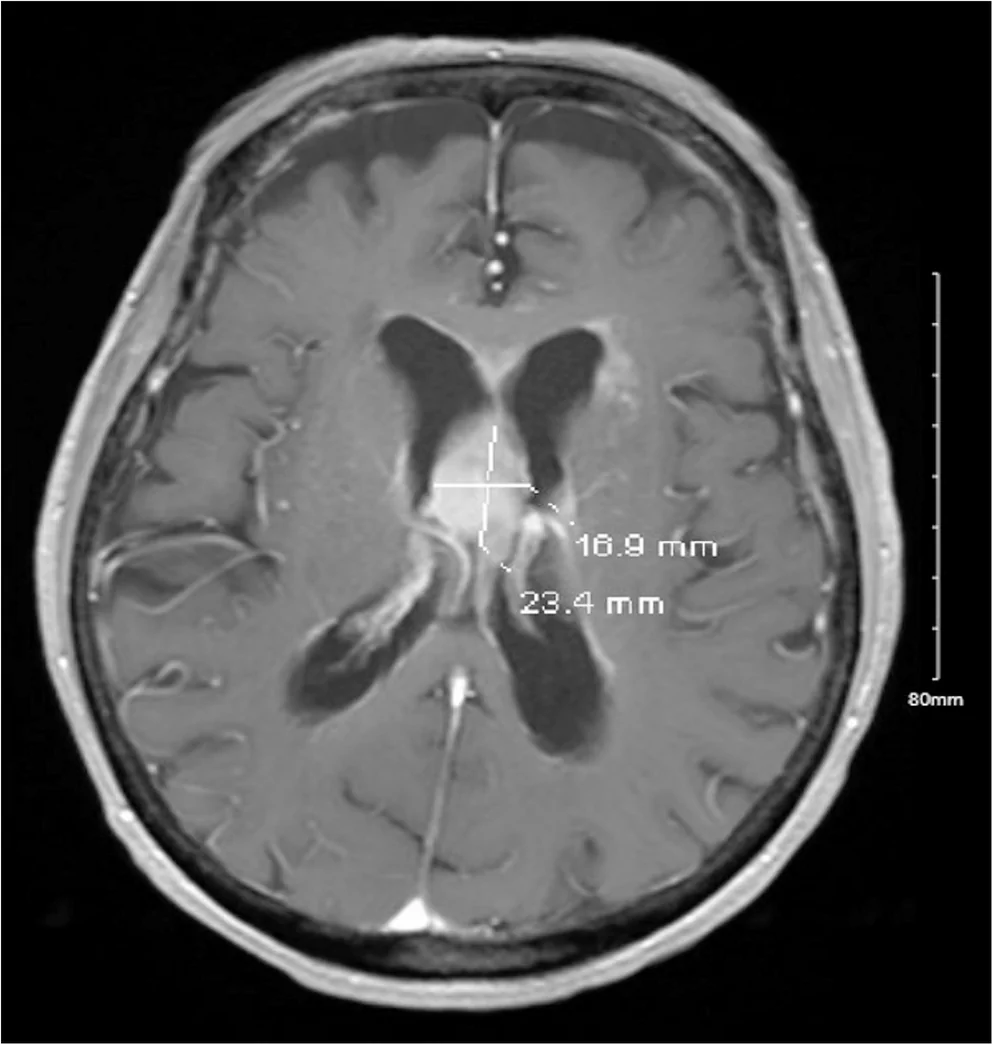
T1-weighted MRI showing a 2.3 × 1.7 × 2.4 cm enhancing tumor along the septum pellucidum extending between the frontal horns of the lateral ventricles with abnormal interventricular enhancement

To further characterize the lesion, contrast-enhanced MRI of the brain was obtained, revealing a 2.3 × 1.7 × 2.4 cm enhancing mass along the septum pellucidum, extending between the frontal horns of the lateral ventricles with abnormal interventricular enhancement (Fig. 2).

**Fig. 3:**
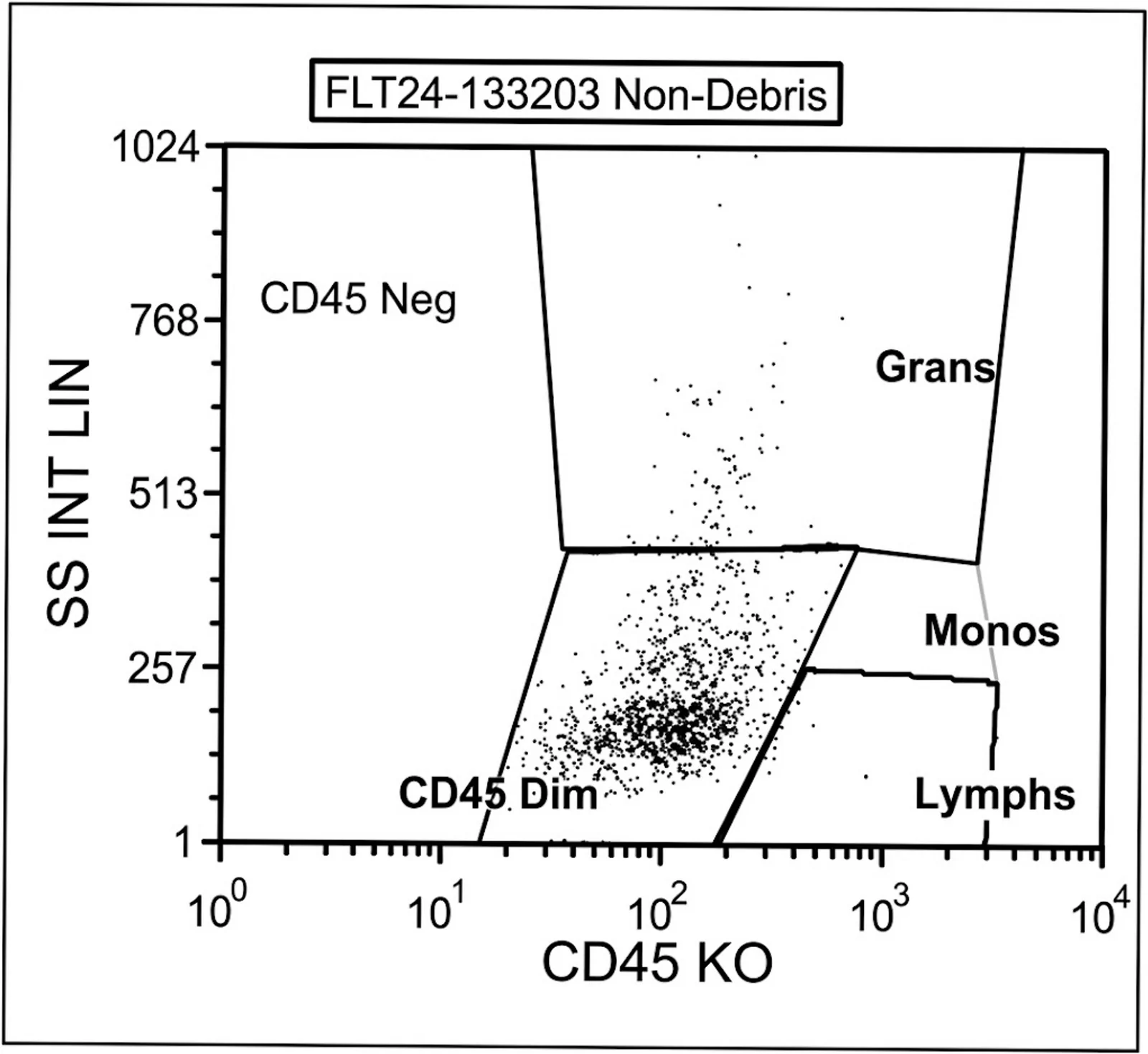
Flow cytometry indicating a 50% population of CD34 + blasts with 82% specimen viability and 0.02 million cell yield, obtained from an external source and enhanced for clarity

Given the surgically challenging location, a tissue biopsy was not performed. Instead, a lumbar puncture was performed to differentiate the lesion from other primary CNS malignancies and to rule out a localized AML relapse. CSF analysis revealed pleocytosis (236 WBC/µL) with 53% mononuclear cells and elevated protein (83 mg/dL). Flow cytometry of the CSF demonstrated a 50% population of CD34 + blasts, which uniquely matched the immunophenotype of the patient’s prior biopsy-confirmed AML diagnosis (Fig. 3).

While primary CNS malignancies, such as high-grade gliomas, were considered on the differential diagnosis due to overlapping features of midline localization and mass effect, the tumor’s well-circumscribed borders and strong, homogenous contrast enhancement were atypical for a primary glial tumor. Additionally, the absence of peripheral blasts or signs of systemic relapse combined with a CSF flow cytometry profile that precisely replicated the patient’s prior CD34 + blast immunophenotype established the diagnosis of isolated intracranial MS (IMS).

A full-body PET-CT scan to screen for additional extramedullary MS was deferred. This decision was based on the patient’s poor prognosis, the lack of an inpatient PET-CT at the facility, and the understanding that identifying additional asymptomatic extramedullary lesions would not alter clinical management or change her prognosis. Due to the patient’s advanced age and the invasive nature of potential therapies required to treat her relapse, the patient and her family opted for palliative care. The patient was transitioned to hospice care and died two months later.

## Discussion

IMS is an extremely rare clinical entity, primarily described in case reports [3–5, 7–12]. One of the largest cohorts, published from Denmark, reported IMS in approximately 0.4% of MS cases [13]. IMS typically involves the brain parenchyma or dura and may present with multiple lesions. Uncommon locations include the temporal lobe (10.3%), cerebellum (10.3%), and falcine/parasagittal regions (10.3%) [6]. Involvement of the septum pellucidum is exceedingly rare, and a review of the indexed literature reveals no prior documented cases of an isolated IMS arising strictly from this location.

Imaging plays a pivotal role in identifying the extent of IMS and differentiating IMS from hemorrhage, primary CNS malignancies, or dural metastases. Imaging findings are often nonspecific, but several patterns have emerged. IMS usually presents as isodense or hyperdense on non-contrast CT compared to isointense or hypointense on T1- and T2-weighted MRI sequences [4, 5]. In previous case reports describing IMS, both imaging modalities typically display well-circumscribed lesions with homogenous enhancement [4, 5, 7, 11–12]. Some reports discuss the presence of vasogenic edema surrounding the tumors, which can add to diagnostic uncertainty [4, 7, 10, 12]. Overall, these imaging findings correlate with our patient’s CT and MRI, aiding in the initial diagnosis of IMS and helping to differentiate it from other CNS tumors or hemorrhage.

Clinical symptoms from IMS typically emerge from mass effect, localized tissue compression, or increased intracranial pressure. In other case reports, patients frequently present with progressive headache, visual deficits, focal neurological impairments, or seizures depending on the lesion’s exact location [3–5, 7–12]. In this case, the tumor’s mass effect on the septum pellucidum likely disrupted limbic pathways, contributing to the patient’s personality changes and hallucinations, a rare presentation of IMS. The septum pellucidum is part of the limbic system and interacts closely with the hypothalamus and hippocampus, playing a role in emotional regulation and memory integration. Abnormalities in this region, such as cavum septum pellucidum, have been associated with increased rates of bipolar disorder, schizophrenia, obsessive-compulsive disorder, and post-traumatic stress disorder [14]. Additionally, structural midline compression or altered ventricular pressure could indirectly disrupt secondary vision pathways, triggering the complex hallucinations seen in this patient [15].

Several established risk factors are associated with CNS relapse in AML, including extramedullary disease at diagnosis, monocytic differentiation, chromosome 16 or 11 abnormalities, FLT3 mutations, and KMT2A rearrangements, none of which were present in this patient. Furthermore, the mutations identified in this patient via NGS, including TET2, EZH2, ASXL1, BCOR, and STAG2, have not been specifically associated with increased IMS risk [16].

The timing of relapse is notable as well. A systematic review by Lee et al., including 99 patients from 82 studies, found that approximately 62% of IMS cases represented the first manifestation of AML relapse, with some occurring up to 10 years after remission [6, 17]. In this case, the patient remained in systemic remission for 5 years while on azacitidine/venetoclax, a notable outcome given that the 5-year survival for AML patients older than 60 years is approximately 5–15% [18, 19]. However, azacitidine and venetoclax have limited penetration across the blood–brain barrier, which may explain the development of isolated CNS disease despite systemic control [20, 21]. Therefore, new neuropsychiatric symptoms in patients with prior AML should prompt evaluation for CNS involvement. While tissue biopsy remains the gold standard [22], this case illustrates that CSF analysis with flow cytometry can provide a definitive diagnosis in surgically high-risk cases. By matching the exact immunophenotype of the primary malignancy, flow cytometry removed the need for an invasive procedure, though broad immunophenotypic testing is required to account for marker variability [6, 22].

There are no standardized treatment guidelines for IMS. Options include chemotherapy and radiotherapy, given the tumor’s radiosensitivity, which can aid in shrinking tumor burden or alleviating cranial nerve deficits [2, 4, 6, 19]. Reported regimens include etoposide and cytarabine following radiation, as well as enasidenib in select cases [20, 21, 23]. Surgical resection via either partial or gross total resection may also be considered, especially for patients with progressive neurological deterioration or acute mass effect [6, 7, 17]. Surgery also provides tissue for histopathological diagnosis [5, 12]. Meta-analytic data compiling historical cases show varied survival timeline, but localized approaches combined with early, aggressive systemic therapy generally yield superior survival advantages compared to localized treatment alone [6]. If treatment is provided, many case reports document full recovery with long-term remission [3–5, 7–12], though regular clinical and neuroimaging follow-up is necessary due to the risk of recurrence or systemic relapse. In this case, a palliative approach was chosen due to patient preference, and the patient ultimately died without treatment.

Clinicians should maintain high suspicion for CNS myeloid sarcoma in patients with hematologic malignancies who present with new neuropsychiatric symptoms, regardless of remission status. Early diagnosis relies heavily on a high index of suspicion and imaging, particularly MRI. In some cases, biopsy may be necessary for a definitive diagnosis, but less invasive diagnostic tools like lumbar puncture and CSF flow cytometry can be especially valuable in high-risk cases. A patient-centered approach is essential in guiding treatment decisions, particularly in elderly or frail individuals.

## Data Availability

No datasets were generated or analysed during the current study.

## Code availability

Data sharing is not applicable to this article as no datasets were generated or analyzed.
